# Preoperative %p2PSA and Prostate Health Index Predict Pathological Outcomes in Patients with Prostate Cancer Undergoing Radical Prostatectomy

**DOI:** 10.1038/s41598-020-57618-2

**Published:** 2020-01-21

**Authors:** Yung-Ting Cheng, Chao-Yuan Huang, Chung-Hsin Chen, Shih-Ting Chiu, Jian-Hua Hong, Yeong-Shiau Pu, Shih-Ping Liu, Yu-Chuan Lu, Yi-Kai Chang, Hong-Chiang Chang, Kuo-How Huang, Yuan-Ju Lee, Po-Ming Chow, I-Ni Chiang, Shih-Chun Hung, Chih-Hung Chiang

**Affiliations:** 1 Department of Urology, National Taiwan University Hospital, Taipei, Taiwan; 2 Department of Urology/Medical Research and Education, Taipei Veterans General Hospital, Yuan-Shan/Su-Ao Branch, Yi-Lan, Taiwan; 3 Department of Nursing, Cardinal Tien Junior College of Healthcare and Management, New Taipei City, Taiwan

**Keywords:** Tumour biomarkers, Tumour biomarkers, Prostate

## Abstract

To evaluate the predictive accuracy of the %p2PSA and prostate health index (PHI) in predicting aggressive pathological outcomes in patients with prostate cancer (PCa) undergoing radical prostatectomy (RP), we enrolled 91 patients with organ-confined PCa who were treated with robot-assisted RP. p2PSA levels and the PHI were investigated for their ability to predict pathological results. The %p2PSA and PHI were both significantly higher in patients with ≥pT3 disease, high-risk disease, positive surgical margin, or seminal vesical invasion (SVI). In univariable analysis, p2PSA derivatives were significant predictors of the presence of ≥pT3 disease, high-risk disease, positive surgical margin, and SVI. To predict adverse pathological outcomes at a sensitivity of 90%, p2PSA derivatives had higher specificity than standard PSA derivatives. In multivariable analysis, additional increases in the area under the receiver operating characteristic curve (AUC) were observed with the %p2PSA and PHI for ≥pT3 disease, high-risk disease, and positive surgical margin (8.2% and 2.7%, 6.2% and 4.1%, and 8.6% and 5.4%, respectively). A PHI ≥61.26 enhanced the predictive accuracy of the model for SVI by increasing the AUC from 0.624 to 0.819 (p = 0.009). The preoperative %p2PSA and PHI accurately predict adverse pathological results and are useful for decision-making.

## Introduction

In patients with prostate cancer (PCa), it is vital to avoid overtreatment and procedural complications. Treatment options for localized PCa including active surveillance, radical prostatectomy (RP), and radiation therapy depend on the aggressiveness of the disease^1^. However, discrepancies often exist between the clinical cancer staging and pathological staging. Approximately half of patients with clinically low-risk PCa at transrectal ultrasonography-guided biopsy of the prostate (TRUSP biopsy) actually have a Gleason score (GS) ≥7 or ≥pathological T3 disease in the final RP pathology^2,3^.

Multiple preoperative predictive nomograms have been validated for the prediction of pathological outcomes at RP. However, the applicability of these nomograms is limited in the clinical setting due to their difficult accessibility and complexity^4^. Therefore, magnetic resonance imaging (MRI) has been increasingly used as an alternative diagnostic tool before prostate biopsy or via MRI-targeted biopsy to better identify clinically significant cancer with a GS ≥7^5^. Correct assessment of the local staging by MRI is still being researched. However, the utility of MRI in local cancer staging is limited by its poor sensitivity for detecting extracapsular extension and seminal vesical invasion (SVI)^6^. Accordingly, we need an accurate and convenient biomarker for preoperatively predicting adverse pathological characteristics and determining who might benefit the most from surgery. Such a biomarker would help physicians in decision-making and to predict prognosis before any intervention.

Total prostate-specific antigen (tPSA) consists of complexed PSA and free PSA (fPSA). Of fPSA forms, p2PSA is one of the isoforms of proPSA, which is considered a promising biomarker that is more cancer-associated^7^. The prostate health index (PHI), developed by Beckman Coulter, Inc., is a mathematical formula combining three biomarkers as follows: (p2PSA/fPSA) × √tPSA. Numerous studies have shown that the %p2PSA and PHI are the most accurate predictors of PCa at the initial prostate biopsy^8–10^. Meanwhile, the %p2PSA and PHI are able to identify aggressive PCa with GS ≥7 prior to TRUSP biopsy and predict unfavorable cancer characteristics at the final pathology from RP^11,12^. However, a lack of a reference range of the %p2PSA and PHI for predicting adverse pathological results limits their clinical utility. In the present study, we aimed to validate the application of the preoperative %p2PSA and PHI to predict adverse pathological outcomes after RP and examine the accuracy of the cut-off values.

## Results

The basic characteristics of the study population are listed in (Table 1**)**. Of the 91 patients, the median age was 64 years (IQR, 60–67). The median preoperative tPSA, fPSA, %fPSA, and PSA density were 10.9 ng/ml, 1.2 ng/ml, 12.1%, and 0.4 ng/ml^2^, respectively. The median preoperative p2PSA, %p2PSA, and PHI were 18.9 pg/ml, 1.9%, and 54.6, respectively. Eventually, 31 (36.3%) and 82 (92.1%) patients were diagnosed with ≥pT3 and pathological GS ≥7 disease, respectively. In addition, 26 patients (28.6%) had an upgraded GS, from GS ≤6 at initial biopsy to a GS sum ≥7 from RP specimens. Overall, 36 patients (39.6%) were classified as having high-risk disease.

**Table 1:** Basic characteristics.

Variables, median	n = 91 patients
Age, years (IQR)	64 (60, 67)
Abnormal DRE, n (%)	35 (38.5%)
**Prior to radical prostatectomy**	
Prostate volume, ml (IQR)	30.5 (23.7, 41.3)
Prostate weight, g (IQR)	35.2 (27.6, 46.4)
tPSA, ng/ml (IQR)	10.9 (7.5, 16.6)
fPSA, ng/ml (IQR)	1.2 (0.7, 2.1)
%fPSA (IQR)	12.1 (8.0, 16.1)
p2PSA, pg/ml (IQR)	18.9 (11.9, 31.5)
%p2PSA (IQR)	1.9 (1.2, 2.3)
PHI (IQR)	54.6 (36.2, 79.2)
PSAD, ng/ml^2^ (IQR)	0.4 (0.2, 0.6)
**Clinical cancer stage, n (%)**	
≤T1c	53 (58.2%)
T2a-2c	38 (41.8%)
**GS at TRUSP biopsy, n (%)**	
≤6	30 (33.0%)
7	40 (44.0%)
≥8	21 (23.1%)
Percentage of positive cores, % (IQR)	20 (10, 40)
Maximum % of positive cores, (IQR)	40 (16, 80)
**Pathological results of radical prostatectomy**	
Pathological stage, n (%)	
pT0	2 (2.2%)
pT2	58 (63.7%)
pT3	30 (35.2%)
pT4	1 (1.1%)
**Pathological GS, n (%)**	
≤6	7 (7.9%)
7	66 (74.2%)
≥8	16 (18.0%)
Positive lymph node, n (%)	10 (11.9%)
Positive surgical margin, n (%)	43 (47.3%)
Upgrade of GS compared with the biopsy, n (%)	26 (28.6%)
High-risk disease, n (%)	36 (39.6%)

IQR: interquartile range; DRE: digital rectal examination; PSA: prostate specific antigen; tPSA = total PSA; fPSA = free PSA; %fPSA = percentage of free to total PSA; p2PSA: [−2]pro PSA; %p2PSA = (p2PSA/fPSA × 1000) × 100; PHI: Prostate Health Index; PSAD: PSA density; GS: Gleason score; TRUSP: transrectal ultrasonography of the prostate.

**Figure 1:**
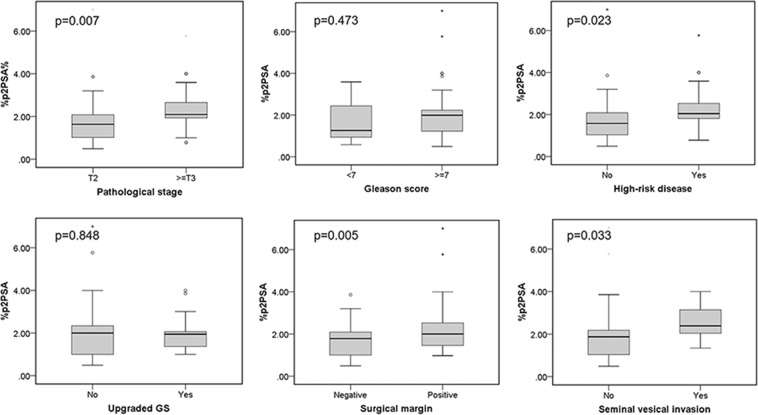
%p2PSA relative to pathological cancer stage, Gleason score, high-risk disease, upgraded Gleason score, surgical margin status, seminal vesical invasion. GS: Gleason score; p2PSA: [−2]pro PSA; p2PSA% = percentage of p2PSA to fPSA ratio; * = Extreme outliers; ° = mild outliers.

**Figure 2:**
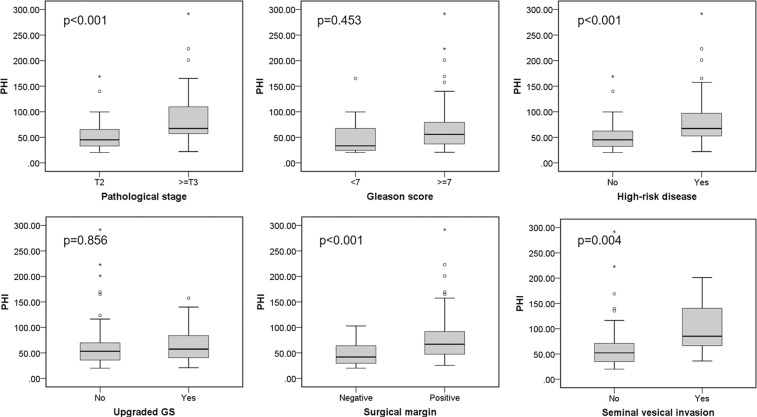
PHI relative to pathological cancer stage, Gleason score, high-risk disease, upgraded Gleason score, surgical margin status, seminal vesical invasion. GS: Gleason score; PHI: Prostate Health Index; * = Extreme outliers; ° = mild outliers.

As shown in (Figs. 1 and 2**)**, the %p2PSA and PHI were significantly higher in patients with ≥pT3 disease, high-risk disease, positive surgical margin, and presence of SVI (%p2PSA: p = 0.007, 0.023, 0.005, and 0.033, respectively; PHI: p < 0.001, <0.001, <0.001, and 0.004, respectively). In the univariable analysis, the p2PSA, %p2PSA, and PHI were accurate parameters for predicting ≥pT3 disease, high-risk disease, and a positive surgical margin (Table 2). The PHI was the only significant predictor of SVI (odds ratio [OR]: 1.01, 95% confidence interval [CI] 1.00–1.03, p = 0.014). Meanwhile, tPSA was also a predictor of ≥pT3 disease, high-risk disease, and positive surgical margin. On the other hand, GS ≥7 at TRUSP biopsy was a predictor of ≥pT3 disease and high-risk disease.

**Table 2:** Univariable analyses to predict pathological outcomes in patients who underwent radical prostatectomy.

	Cancer stage ≥ pT3		Gleason score ≥ 7		High-risk disease	
	OR (95% CI)	p value	OR (95% CI)	p value	OR (95% CI)	p value
Age	1.01 (0.93, 1.11)	0.754	1.18 (1.02, 1.38)	0.031	1.01 (0.92, 1.10)	0.866
Prostate volume	0.99 (0.97, 1.01)	0.428	0.98 (0.96, 1.01)	0.170	0.99 (0.96, 1.01)	0.274
Biopsy GS ≥7	**5.91 (1.59, 22.06)**	**0.008**	2.24 (0.59, 8.44)	0.233	**5.17 (1.75, 15.27)**	**0.003**
tPSA	**1.07 (1.01, 1.14)**	**0.028**	1.02 (0.93, 1.13)	0.614	**1.07 (1.01, 1.14)**	**0.030**
fPSA	1.42 (0.92, 2.18)	0.109	0.86 (0.48, 1.54)	0.606	1.41 (0.92, 2.16)	0.117
%fPSA	0.95 (0.87, 1.03)	0.199	0.91 (0.81, 1.01)	0.078	0.94 (0.87, 1.02)	0.157
p2PSA	**1.03 (1.01, 1.06)**	**0.005**	1.01 (0.98, 1.03)	0.702	**1.03 (1.01, 1.06)**	**0.007**
%p2PSA	**1.79 (1.08, 2.95)**	**0.023**	1.38 (0.61, 3.10)	0.438	**1.62 (1.01, 2.61)**	**0.047**
PHI	**1.02 (1.01, 1.04)**	**0.002**	1.01 (0.99, 1.03)	0.452	**1.02 (1.01, 1.04)**	**0.003**
	**Upgraded GS sum**		**Positive surgical margin**		**Seminal vesical invasion**	
	**OR (95% CI)**	**p value**	**OR (95% CI)**	**p value**	**OR (95% CI)**	**p value**
Age	1.06 (0.96, 1.18)	0.213	1.00 (0.92, 1.09)	0.964	0.99 (0.87, 1.13)	0.874
Prostate volume	1.01 (0.99, 1.03)	0.291	0.98 (0.96, 1.01)	0.182	1.00 (0.96, 1.03)	0.839
Biopsy GS ≥7	0.00 (0.00, 0.03)	<0.001	1.55 (0.64, 3.76)	0.333	2.42 (0.49, 12.00)	0.278
tPSA	1.01 (0.96, 1.07)	0.654	**1.11 (1.03, 1.18)**	**0.004**	1.07 (1.00, 1.15)	0.055
fPSA	1.23 (0.80, 1.89)	0.337	1.34 (0.87, 2.05)	0.183	1.36 (0.80, 2.32)	0.250
%fPSA	1.00 (0.92, 1.09)	0.987	0.94 (0.86, 1.01)	0.098	0.92 (0.81, 1.06)	0.247
p2PSA	1.01 (0.99, 1.02)	0.421	**1.04 (1.01, 1.07)**	**0.006**	1.02 (1.00, 1.03)	0.085
%p2PSA	0.96 (0.62, 1.50)	0.861	**2.06 (1.18, 3.60)**	**0.011**	1.55 (0.95, 2.55)	0.080
PHI	1.00 (0.99, 1.01)	0.858	**1.03 (1.01, 1.05)**	**0.001**	**1.01 (1.00, 1.03)**	**0.014**

CI: confidence interval; GS: Gleason score; OR: odds ratio; PHI: Prostate Health Index; PSA: prostate specific antigen; tPSA: total PSA; fPSA: free PSA; %fPSA = percentage of free to total PSA; p2PSA: [−2]pro PSA; %p2PSA = (p2PSA/fPSA × 1000) × 100.

At a sensitivity of 90% to predict the presence of ≥pT3 cancer, high-risk disease, positive surgical margin, or SVI, the %p2PSA or PHI had a higher specificity in comparison with tPSA (Table 3). In this setting, the most appropriate cut-off values for %p2PSA were ≥1.21, ≥1.12, and ≥1.17 for the presence of ≥pT3 cancer, high-risk disease, and positive surgical margin, respectively. In contrast, the most appropriate cut-off values for PHI were determined to be ≥33.92, ≥33.92, ≥33.92, and ≥61.26 for the presence of ≥pT3 cancer, high-risk disease, positive surgical margin, and SVI, respectively.

**Table 3:** The cut-off values of p2PSA derivatives and the PHI in predicting ≥pT3 cancer, high-risk disease, a positive surgical margin, and seminal vesical invasion at a sensitivity of 90%.

Outcome	PSA derivatives	Cut-off	Specificity
Cancer stage ≥pT3	tPSA	≥5.95	16.7%
	p2PSA	≥10.51	15.0%
	%p2PSA	≥1.21	30.0%
	PHI	≥33.92	26.7%
High-risk disease	tPSA	≥5.95	18.2%
	p2PSA	≥10.51	16.4%
	%p2PSA	≥1.12	29.1%
	PHI	≥33.92	29.1%
Positive surgical margin	tPSA	≥5.34	16.7%
	p2PSA	≥10.46	14.6%
	%p2PSA	≥1.17	33.3%
	PHI	≥33.92	31.3%
Seminal vesical invasion	tPSA	≥7.22	25.0%
	PHI	≥61.26	66.3%

PSA: prostate specific antigen; tPSA: total PSA; p2PSA: [−2]pro PSA; %p2PSA = (p2PSA/fPSA × 1000) × 100; PHI: Prostate Health Index.

In the multivariable analysis, base models for each predictive end point were selected according to the univariable analytical results. Thus, age, prostate volume, tPSA, and biopsy GS ≥7 were selected as the base model for a cancer stage ≥pT3 and high-risk disease, whereas age, prostate volume, and tPSA acted as the base model for a positive surgical margin and SVI (Supplementary Table). We separately tested the predictive accuracy of the p2PSA, %p2PSA, or PHI by adding them individually to the base model. For predicting ≥pT3 cancer, a %p2PSA ≥1.21 had a statistically significant OR of 5.41 (95% CI 1.33–22.04, p = 0.019) and increased the AUC from 0.687 to 0.768 (p = 0.073). To predict the presence of high-risk disease, a %p2PSA ≥1.12 and PHI ≥33.92 had significant ORs of 6.94 (95% CI 1.66–29.06, p = 0.008) and 4.52 (95% CI 1.08–19.00, p = 0.039), respectively. The addition of the %p2PSA and PHI to the base model increased the AUC for predicting high-risk disease by 0.062 (p = 0.195) and 0.041 (p = 0.104), respectively. For predicting a positive surgical margin, a %p2PSA ≥1.17 and PHI ≥33.92 had significant ORs of 4.04 (95% CI 1.24–13.15, p = 0.020) and 3.91 (95% CI 1.12–13.63, p = 0.032), respectively. The addition of the %p2PSA and PHI increased the AUCs by 0.086 (p = 0.097) and 0.054 (p = 0.156), respectively. A PHI ≥61.26 significantly predicted SVI with an OR of 20.85 (95% CI 2.26–191.91, p = 0.007). Inclusion of the PHI in the base model significantly increased the AUC from 0.624 to 0.819 (p = 0.009).

## Discussion

In this cohort, we confirmed the accuracy of the preoperative %p2PSA and PHI in predicting adverse pathological results for patients with clinically organ-confined PCa undergoing RP. The %p2PSA and PHI had excellent ability to predict the four major pathological outcomes considered: ≥pT3 cancer, high-risk disease, positive surgical margin, and SVI. An additional predictive benefit was provided by the addition of the %p2PSA and PHI to the base model in the multivariable analysis, with particular benefit of the PHI in predicting SVI. There has been no consensus on the most appropriate cut-off value for the %p2PSA and PHI in cancer detection due to the use of different study designs^11^. However, prior studies investigating the predictive value of the %p2PSA and PHI in patients treated with RP did not provide a reference range for clinical utility^8,12–14^. The major strength of the current study is that we offered cut-off values of p2PSA derivatives and tested their predictive accurracy in a multivariable model **(**Table 3 and Supplementary Table**)**. As a result, it will be easier for physicians to have a clear threshold of p2PSA derivatives at clinical application.

A meta-analysis showed that %p2PSA and PHI could detect more aggressive PCa with GS ≥7 at the initial prostate biopsy (AUCs of 0.54 and 0.67 for %p2PSA and PHI, respectively)^11^. In patients indicated for the first prostate biopsy or repeated biopsy, NCCN guidelines suggest that a PHI >35 indicates a higher probability of high-grade PCa. Such an association with cancer aggressiveness has been extended to patients with PCa undergoing active surveillance. Tosoian *et al*.^15^ revealed that both baseline and longitudinal %p2PSA and PHI provided outstanding predictive value in biopsy reclassification and upgraded the GS in men under active surveillance. To further extend these results, examination of the relationship between p2PSA derivatives and the final pathology is warranted.

Although our findings failed to confirm the prediction of pathological GS ≥7 PCa, the ability of p2PSA derivatives to predict aggressive PCa was still confirmed. The potential causes of the failure to predict a pathological GS ≥7 include the small sample size of our cohort and the high proportion of patients (82 of 92; 89.1%) with pT3 disease or a GS ≥7. However, our cohort could not represent a comprehensive patient group with organ-confined PCa due to treatment indications. Secondly, the results may suggest that p2PSA derivatives better correlate with the extent of cancer invasion than the cancer grade. Heidegger *et al*.^16^ revealed that the highest p2PSA was seen in patients with a GS ≥8 at RP and the lowest in those with a GS ≤6. A significant difference was seen in p2PSA values between a GS ≥8 and GS ≤7 (p < 0.01). Guazzoni *et al*.^17^ confirmed that the %p2PSA and PHI were accurate biomarkers of pT3 disease, a pathological GS ≥7, an upgraded GS, and tumor volume < 0.5 ml in men undergoing RP. Another multicenter study by Fossati *et al*.^12^ also supported their accurate prediction of pT3 disease and/or a pathological GS ≥7. However, the %p2PSA and PHI seemed to provide slight benefit to the traditional predictive models. The increase in the AUC with the PHI for predictive accuracy was actually low for pT3 cancer (2.0–2.5%) and a pathological GS ≥7 (3–6%).

Previous reports have indicated that the reference ranges of biomarkers should be adjusted for different ethnic groups. Rhodes *et al*.^18^ showed that p2PSA derivatives were slightly higher in black men than in white men. Lower PHI values with a higher AUC for cancer detection at the initial TRUSP biopsy were observed in an Asian series compared with European studies^10,19^. At a cut-off range of the PHI between 35 and 55, Asian men had a lower detection rate of PCa and high-grade PCa than European men. Lower cut-off values were thus applied to Asian men for detecting PCa at the initial TRUSP biopsy^19^. Chiu *et al*. showed that the %p2PSA and PHI in Asian men had a higher AUC increase over the base model in predicting pT3 or a pathological GS ≥7 than in the Western studies (7.9% and 7.2% vs. 1.2% and 2.3%, respectively)^12,13^. The net clinical benefit of PHI in predicting pT3 or a pathological GS ≥7 was demonstrated in decision curve analysis when the threshold probability ranged between 20% and 45%^13^. Our findings seem to be consistent with these results, with a PHI at a lower cut-off value of 33.92 predicting the presence of ≥pT3 PCa, high-risk disease, and a positive surgical margin.

Several novel biomarkers have been simultaneously compared with the PHI for predicting pathological results from RP, such as prostate cancer antigen 3 (PCA3) or TMPRSS2:ERG fusions^20,21^. Both the PHI and PCA3 significantly increased the predictive accuracy of the base model for extracapsular extension, whereas the PHI added only incremental value for predicting a pathological GS ≥7 and SVI^20^. Tallon *et al*.^21^ suggested that the PHI was a more reliable biomarker than the other two markers to predict a pathological GS ≥7. Both the PHI and the TMPRSS2:ERG test could significantly predict extracapsular extension. A notable 14% increase in the AUC was seen when these three biomarkers were combined with the base model.

The performance of the PHI and MRI in predicting significant PCa after RP were compared by Porpiglia *et al*.^22^. A 4% increase in the AUC over the base model was added by the PHI (AUC = 0.75, p < 0.01) and a 7% increase in the AUC over the base model was seen with MRI (AUC = 0.78, p < 0.01). Nevertheless, the optimal sequences for combining serum markers and imaging studies in the clinical setting should be further investigated. Further cost-effectiveness analysis should also be taken into consideration, given that, compared with the PHI, MRI is less suitable in many ways; for instance, it is more expensive, has a greater demand for professional radiological interpretation, and has more limited equipment availability^23^.

Several limitations are found in our study. Although the small sample size of our cohort may limit the statistical significance, we demonstrated the outstanding predictive accuracy of the %p2PSA and PHI. Second, the patient group included in this study could not be used as a surrogate of patients with organ-confined PCa. A selection bias may exist because surgical indications are affected by factors such as patients’ decisions, age, or comorbidities. Finally, the pathological specimens were reviewed by different urogenital pathologists instead of via centralized evaluation.

In conclusion, the %p2PSA and PHI accurately predict aggressive pathological features in RP specimens, including the presence of ≥pT3 cancer, high-risk disease, positive surgical margin, and SVI. The cut-off values of p2PSA derivatives improve their application in clinical practice. In particular, a PHI ≥61.26 significantly increases the predictive accuracy of the model for identifying the presence of SVI.

## Methods

Between February 2017 and June 2018, 91 men with biopsy-proven clinically organ-confined PCa who underwent robot-assisted RP were prospectively enrolled from the National Taiwan University Hospital, a tertiary medical institution. Clinical data were obtained, including age, digital rectal examination, prostate volume, prostate weight, PSA derivatives, GS from TRUSP biopsy, number of positive biopsy cores, and percentage of positive biopsy cores. Cancer staging was completed with bone scintigraphy and MRI. Exclusion criteria included any possible factors that might alter PSA values: (1) active urinary tract infection; (2) use of 5-alpha reductase inhibitors such as finasteride or dutasteride; (3) preoperative androgen-deprivation therapy; and (4) transurethral resection of the prostate prior to the RP.

The novel biomarkers p2PSA, %p2PSA [(p2PSA/fPSA × 1000) × 100], and PHI were compared with the widely accepted standard tests: tPSA, fPSA, and %fPSA. Blood samples were drawn prior to the RP after informed consent was obtained from patients. Within 3 hours of blood collection, the serum samples were processed by centrifugation at 1500 × *g* for 15 minutes and stored at −20 °C until analysis, as reported by Semjonow *et al*.^24^. The blood samples were analyzed with a Beckman Coulter Access 2 immunoassay analyzer (Beckman Coulter Taiwan Inc.) with Beckman Coulter Access Hybritech reagent and calibrators. Specimens from TRUSP biopsy and RP were evaluated by experienced genitourinary pathologists who were blinded to the serum results.

The primary objective of our study focused on investigating the accuracy of the p2PSA, %p2PSA, and PHI in predicting adverse pathological features from RP, specifically: (1) extracapsular disease (pT3), (2) pathological GS ≥7 cancer, (3) high-risk disease (defined as pT3 and/or GS ≥8 based on the risk stratification of the NCCN guidelines in 2018), (4) upgrading of the GS sum from ≤6 at biopsy to ≥7 at RP specimen analysis, (5) positive surgical margin, and (6) SVI.

Statistical analyses were conducted with SPSS version 22.0 (IBM Corp, Inc., Chicago, IL, USA). The median and interquartile range (IQR) are presented for non-nominal variables. First, univariable analysis was used to test the ability of the parameters to predict pathological outcomes. Then, the cut-off values of each significant PSA or p2PSA derivative were selected at 90% sensitivity based on the area under the receiver operating characteristic curve (AUC). Finally, we examined the cut-off values of p2PSA derivatives separately in multivariable logistic regression models for their ability to predict aggressive pathological results. The AUCs of different predictive models were compared separately with the basic model. A two-sided p < 0.05 was considered statistically significant.

The present cohort was supported by the Institutional Review Board and Research Ethics Committee of National Taiwan University Hospital (Approval Code: 201612091RIPD). All methods were performed in accordance with the relevant guidelines and regulations. The informed consent was obtained from all participants and/or their legal guardians. The biomarker reagents were sponsored by Beckman Coulter Taiwan Inc., which had no participation in the study design or statistical analysis and was blinded to the results.

## Supplementary information

Supplementary table.
