# Supplementary material for: Preoperative %p2PSA and Prostate Health Index Predict Pathological Outcomes in Patients with Prostate Cancer Undergoing Radical Prostatectomy
Source: Sci Rep. 2020 Jan 21;10:776. doi: 10.1038/s41598-020-57618-2 (PMC6972898; doi:10.1038/s41598-020-57618-2)
Supplement: Supplementary file 1 — Supplementary table. [file 41598_2020_57618_MOESM1_ESM.docx]

**Original article**

**Title Page**

**Title**

**Preoperative %p2PSA and Prostate Health Index Predict Pathological Outcomes in Patients with Prostate Cancer Undergoing Radical Prostatectomy**

Yung-Ting Cheng^1^, Chao-Yuan Huang^1^, Chung-Hsin Chen^1^, Shih-Ting Chiu^1^, Jian-Hua Hong^1^, Yeong-Shiau Pu^1^, Shih-Ping Liu^1^, Yu-Chuan Lu^1^, Yi-Kai Chang^1^, Hong-Chiang Chang^1^, Kuo-How Huang^1^, Yuan-Ju Lee^1^, Po-Ming Chow^1^, I-Ni Chiang^1^, Shih-Chun Hung^1^, Chih-Hung Chiang^1,2,3^

^1^Department of Urology, National Taiwan University Hospital, Taipei, Taiwan

^2^Department of Urology/Medical Research and Education, Taipei Veterans General Hospital, Yuan-Shan/Su-Ao Branch, Yi-Lan, Taiwan

^3^Department of Nursing, Cardinal Tien Junior College of Healthcare and Management, New Taipei City, Taiwan

**Correspondence:**

**Chih-Hung Chiang**

Department of Urology, National Taiwan University Hospital, Taipei, Taiwan

Department of Urology/Medical Research and Education, Taipei Veterans General Hospital, Yuan-Shan/Su-Ao Branch, Yi-Lan, Taiwan

Department of Nursing, Cardinal Tien Junior College of Healthcare and Management, New Taipei City, Taiwan

Postal Address:

Department of Urology, National Taiwan University Hospital, No.7, Zhongshan S. Rd., Zhongzheng Dist., Taipei City 100, Taiwan (R.O.C.).

E-mail: [guchiang@gmail.com](mailto:guchiang@gmail.com)

Tel: +886-2- 23123456 ext. 65238

Fax: +886-2-2321-9145

**First Author: Yung-Ting Cheng**

Department of Urology, National Taiwan University Hospital, No.7, Zhongshan S. Rd., Zhongzheng Dist., Taipei City 100, Taiwan (R.O.C.).

E-mail: [royaloftheworld@yahoo.com.tw](mailto:royaloftheworld@yahoo.com.tw)

**Chao-Yuan Huang**

Department of Urology, National Taiwan University Hospital, No.7, Zhongshan S. Rd., Zhongzheng Dist., Taipei City 100, Taiwan (R.O.C.).

E-mail: [cyh540909@gmail.com](mailto:cyh540909@gmail.com)

**Chung-Hsin Chen**

Department of Urology, National Taiwan University Hospital, No.7, Zhongshan S. Rd., Zhongzheng Dist., Taipei City 100, Taiwan (R.O.C.).

E-mail: mufasachen@gmail.com

**Shih-Ting Chiu**

Department of Urology, National Taiwan University Hospital, No.7, Zhongshan S. Rd., Zhongzheng Dist., Taipei City 100, Taiwan (R.O.C.).

E-mail: stin0429@gmail.com

**Jian-Hua Hong**

Department of Urology, National Taiwan University Hospital, No.7, Zhongshan S. Rd., Zhongzheng Dist., Taipei City 100, Taiwan (R.O.C.).

E-mail: luckymonkey999@hotmail.com

**Yeong-Shiau Pu**

Department of Urology, National Taiwan University Hospital, No.7, Zhongshan S. Rd., Zhongzheng Dist., Taipei City 100, Taiwan (R.O.C.).

E-mail: [yspu@ntu.edu.tw](mailto:yspu@ntu.edu.tw)

**Shih-Ping Liu**

Department of Urology, National Taiwan University Hospital, No.7, Zhongshan S. Rd., Zhongzheng Dist., Taipei City 100, Taiwan (R.O.C.).

E-mail: spliu@ntuh.gov.tw

**Yu-Chuan Lu**

Department of Urology, National Taiwan University Hospital, No.7, Zhongshan S. Rd., Zhongzheng Dist., Taipei City 100, Taiwan (R.O.C.).

E-mail: [charleslu0115@gmail.com](mailto:charleslu0115@gmail.com)

**Yi-Kai Chang**

Department of Urology, National Taiwan University Hospital, No.7, Zhongshan S. Rd., Zhongzheng Dist., Taipei City 100, Taiwan (R.O.C.).

E-mail: changik0115@gmail.com

**Hong-Chiang Chang**

Department of Urology, National Taiwan University Hospital, No.7, Zhongshan S. Rd., Zhongzheng Dist., Taipei City 100, Taiwan (R.O.C.).

E-mail: Changhong@ntu.edu.tw

**Kuo-How Huang**

Department of Urology, National Taiwan University Hospital, No.7, Zhongshan S. Rd., Zhongzheng Dist., Taipei City 100, Taiwan (R.O.C.).

E-mail: [kuohowhuang@gmail.com](mailto:kuohowhuang@gmail.com)

**Yuan-Ju Lee**

Department of Urology, National Taiwan University Hospital, No.7, Zhongshan S. Rd., Zhongzheng Dist., Taipei City 100, Taiwan (R.O.C.).

E-mail: leeyuanju@hotmail.com

**Po-Ming Chow**

Department of Urology, National Taiwan University Hospital, No.7, Zhongshan S. Rd., Zhongzheng Dist., Taipei City 100, Taiwan (R.O.C.).

E-mail: meow1812@gmail.com

**I-Ni Chiang**

Department of Urology, National Taiwan University Hospital, No.7, Zhongshan S. Rd., Zhongzheng Dist., Taipei City 100, Taiwan (R.O.C.).

E-mail: inichiang@gmail.com

**Shih-Chun Hung**

Department of Urology, National Taiwan University Hospital, No.7, Zhongshan S. Rd., Zhongzheng Dist., Taipei City 100, Taiwan (R.O.C.).

E-mail: neashung@gmail.com

**Supplementary table. Multivariable analysis to predict adverse pathological outcomes in patients treated with radical prostatectomy: (a) cancer stage ≥ pT3 disease; (b) high-risk disease; (c) positive surgical margin; and (d) seminal vesical invasion**

|  | | Base model  OR (95% CI); p value | Base model+p2PSA  OR (95% CI); p value | Base model+%p2PSA  OR (95% CI); p value | Base model+PHI  OR (95% CI); p value |
| --- | --- | --- | --- | --- | --- |
| **a. Cancer stage ≥ pT3** | | | | | |
| Age | | 1.02 (0.92, 1.12); 0.752 | 1.01 (0.92, 1.12); 0.804 | 1 (0.9, 1.11); 0.973 | 1.01 (0.92, 1.12) ; 0.798 |
| Prostate volume | | 0.99 (0.97, 1.02); 0.696 | 0.99 (0.97, 1.02); 0.620 | 1 (0.97, 1.03); 0.916 | 1.00 (0.97, 1.02) ; 0.828 |
| tPSA | |  |  |  |  |
|  | <5.95 | 1.00 (Ref) | 1.00 (Ref) | 1.00 (Ref) | 1.00 (Ref) |
|  | ≥5.95 | 2.39 (0.57, 10.04); 0.234 | 1.73 (0.36, 8.38); 0.500 | 3.5 (0.8, 15.25); 0.095 | 1.48 (0.32, 6.97) ; 0.617 |
| Biopsy GS ≥ 7 | | 5.55 (1.68, 18.29); 0.005 | 5.95 (1.79, 19.79); 0.004 | 6.21 (1.82, 21.23); 0.004 | 5.83 (1.74, 19.47) ; 0.004 |
| p2PSA | |  |  |  |  |
|  | <10.51 | - | 1.00 (Ref) | - | - |
|  | ≥10.51 | - | 2.14 (0.43, 10.57); 0.352 | - | - |
| %p2PSA | |  |  |  |  |
|  | <1.21 | - | - | 1.00 (Ref) | - |
|  | ≥1.21 | - | - | 5.41 (1.33, 22.04); **0.019** | - |
| PHI | |  |  |  |  |
|  | <33.92 | - | - | - | 1.00 (Ref) |
|  | ≥33.92 | - | - | - | 3.48 (0.82, 14.75) ; 0.090 |
| AUC of the predictive model | | 0.687 | 0.714 | 0.768 | 0.714 |
| Increase in the AUC for predictive accuracy | | - | 2.7% | 8.2% | 2.7% |
| p value compared with the base model | | - | 0.235 | 0.073 | 0.206 |
| **b. High-risk disease** | | | | | |
| Age | | 1.01 (0.92, 1.11); 0.866 | 1 (0.91, 1.11); 0.938 | 0.99 (0.89, 1.10); 0.848 | 1 (0.91, 1.11); 0.939 |
| Prostate volume | | 0.99 (0.97, 1.02); 0.482 | 0.99 (0.96, 1.01); 0.398 | 1 (0.97, 1.03); 0.946 | 0.99 (0.97, 1.02); 0.620 |
| tPSA | |  |  |  |  |
|  | <5.95 | 1.00 (Ref) | 1.00 (Ref) | 1.00 (Ref) | 1.00 (Ref) |
|  | ≥5.95 | 3.41 (0.81, 14.34); 0.095 | 2.21 (0.46, 10.64); 0.322 | 5.26 (1.19, 23.25); 0.029 | 1.97 (0.42, 9.33); 0.391 |
| Biopsy GS ≥ 7 | | 5.63 (1.85, 17.17); 0.002 | 6.24 (2.02, 19.26); 0.001 | 6.78 (2.11, 21.76); 0.001 | 6.04 (1.94, 18.80); 0.002 |
| p2PSA | |  |  |  |  |
|  | <10.51 | - | 1.00 (Ref) | - | - |
|  | ≥10.51 | - | 2.87 (0.58, 14.1); 0.195 | - | - |
| %p2PSA | |  |  |  |  |
|  | <1.12 | - | - | 1.00 (Ref) | - |
|  | ≥1.12 | - | - | 6.94 (1.66, 29.06); **0.008** | - |
| PHI | |  |  |  |  |
|  | <33.92 | - | - | - | 1.00 (Ref) |
|  | ≥33.92 | - | - | - | 4.52 (1.08, 19.00); **0.039** |
| AUC of the predictive model | | 0.694 | 0.730 | 0.756 | 0.735 |
| Increase in the AUC for predictive accuracy | | - | 3.6% | 6.2% | 4.1% |
| p value compared with the base model | | - | 0.179 | 0.195 | 0.104 |
| **c. Positive surgical margin** | | | | | |
| Age | | 1.01 (0.92, 1.11); 0.821 | 1.01 (0.92, 1.11); 0.846 | 0.99 (0.90, 1.09); 0.817 | 1 (0.91, 1.10); 0.986 |
| Prostate volume | | 0.98 (0.96, 1.01); 0.154 | 0.98 (0.96, 1.01); 0.138 | 0.99 (0.97, 1.01); 0.429 | 0.99 (0.96, 1.01); 0.223 |
| tPSA | |  |  |  |  |
|  | <5.34 | 1.00 (Ref) | 1.00 (Ref) | 1.00 (Ref) | 1.00 (Ref) |
|  | ≥5.34 | 2.02 (0.55, 7.38); 0.289 | 1.69 (0.41, 7.02); 0.470 | 2.87 (0.76, 10.87); 0.120 | 1.35 (0.34, 5.43); 0.670 |
| p2PSA | |  |  |  |  |
|  | <10.46 | - | 1.00 (Ref) | - | - |
|  | ≥10.46 | - | 1.54 (0.35, 6.80); 0.565 | - | - |
| %p2PSA | |  |  |  |  |
|  | <1.17 | - | - | 1.00 (Ref) | - |
|  | ≥1.17 | - | - | 4.04 (1.24, 13.15); **0.020** | - |
| PHI | |  |  |  |  |
|  | <33.92 | - | - | - | 1.00 (Ref) |
|  | ≥33.92 | - | - | - | 3.91 (1.12, 13.63); **0.032** |
| AUC of the predictive model | | 0.553 | 0.569 | 0.639 | 0.607 |
| Increase in the AUC for predictive accuracy | | - | 1.6% | 8.6% | 5.4% |
| p value compared with the base model | | - | 0.515 | 0.097 | 0.156 |
| **d. Seminal vesical invasion** | | | | | |
| Age | | 0.96 (0.83,1.11); 0.585 | - | - | 0.95 (0.81, 1.10); 0.488 |
| Prostate volume | | 0.99 (0.96,1.03); 0.772 | - | - | 1.01 (0.97, 1.06); 0.568 |
| tPSA | |  |  |  |  |
|  | <7.22 | 1.00 (Ref) | - | - | 1.00 (Ref) |
|  | ≥ 7.22 | 3.8 (0.43,34.02); 0.232 | - | - | 1.76 (0.18, 17.51); 0.630 |
| PHI | |  |  |  |  |
|  | <61.26 | - | - | - | 1.00 (Ref) |
|  | ≥61.26 | - | - | - | 20.85 (2.26, 191.91); **0.007** |
| AUC of the predictive model | | 0.624 | - | - | 0.819 |
| Increase in the AUC for predictive accuracy | | - | - | - | 19.5% |
| p value compared with the base model | | - | - | - | **0.009** |
| AUC: area under the receiver operating characteristic curve; CI: confidence interval; GS: Gleason score; OR: odds ratio; PHI: Prostate Health Index; PSA: prostate specific antigen; tPSA: total PSA; fPSA: free PSA; %fPSA=percentage of free to total PSA; p2PSA: [-2]pro PSA; %p2PSA=(p2PSA/fPSA x 1000) x 100; Ref: reference | | | | | |
